# An updated review on probiotics as an alternative of antibiotics in poultry — A review

**DOI:** 10.5713/ab.21.0485

**Published:** 2022-01-21

**Authors:** Muhammad Umar Yaqoob, Geng Wang, Minqi Wang

**Affiliations:** 1 College of Animal Science, Zhejiang University, Key Laboratory of Animal Nutrition and Feed Science (Eastern China), Ministry of Agriculture, Hangzhou 310058, China

**Keywords:** Growth, Immunity, Intestinal Health, Poultry

## Abstract

Antibiotics used to be supplemented to animal feeds as growth promoter and as an effective strategy to reduce the burden of pathogenic bacteria present in the gastro-intestinal tract. However, in-feed antibiotics also kill bacteria that may be beneficial to the animal. Secondly, unrestricted use of antibiotics enhanced the antibiotic resistance in pathogenic bacteria. To overcome above problems, scientists are taking a great deal of measures to develop alternatives of antibiotics. There is convincing evidence that probiotics could replace in-feed antibiotics in poultry production. Because they have beneficial effects on growth performance, meat quality, bone health and eggshell quality in poultry. Better immune responses, healthier intestinal microflora and morphology which help the birds to resist against disease attack were also identified with the supplementation of probiotics. Probiotics establish cross-feeding between different bacterial strains of gut ecosystem and reduce the blood cholesterol level via bile salt hydrolase activity. The action mode of probiotics was also updated according to recently published literatures, i.e antimicrobial substances generation or toxin reduction. This comprehensive review of probiotics is aimed to highlight the beneficial effects of probiotics as a potential alternative strategy to replace the antibiotics in poultry.

## INTRODUCTION

Since they were discovered antibiotics (ABs) have been used for prevention and treatment of infectious diseases and when added at sub-therapeutic level in diet as a growth promoter in many animal species. Antibiotics also performed significant role in the improvement and success of animal husbandry. Chicken is the only species with highest population worldwide reared as food animal and highest concentration with different strains, ABs are used in poultry farming [1]. When ABs are used in any system, they kill the susceptible bacterial leaving behind some resistant strains, which increase in number with the passage of time and might become the cause of resistant genes transfer to other bacteria [2]. These resistant bacteria may transfer from one host to other, directly, or indirectly and lower the effectiveness of drugs and become the cause of antibiotics resistance (ABR), [3]. In this way ABs exhibit negative effects on animals and human when used continuously at sub-therapeutic for long time or used in excess [4]. ABR in bacteria could ether be inherent directly or acquired from other species of bacteria in the environment [5] and develops by different ways such as lowering the permeability of bacterial cell membrane, developing changes in binding sites where the ABs attach in bacteria or/and alteration in enzyme production [6]. There are several bacteria which have developed ABR due to improper use of ABs. *Staphylococcus* species which commonly affect the chicken and cause pododermatitis, staphylococcosis, and septicemia have been developed ABR against β-lactams [7]. Some *Staphylococcus* species also developed ABR against tetracycline and oxacillin [8,9]. Similarly, *Pseudomonas aeruginosa* (*P. aeruginosa*) has developed ABR against carbapenems, quinolones, aminoglycoside, cephalosporins, penicillins and monobactam [10]. *P. aeruginosa* obtained from chicken in Pakistan was 100% resistant to colistin, ceftriaxone, erythromycin, meropenem and ciprofloxacin [11]. In addition, *Escherichia coli* (*E. coli*) is now resistant to most commonly used drug in poultry i.e. tetracycline [12]. Different *Salmonella* spp. have developed ABR against ampicillin, tetracycline, trimethoprim, ciprofloxacin and sulfamethazole [13]. Similarly, *Campylobacter jejuni* and *E. coli* have also been reported to resistant to erythromycin and tetracycline [14].

Keeping in view ABR and other negative effects caused by unrestricted use of ABs, the European Union banned the use of ABs in animals feed in 2006 [15]. Similarly, the US Food and Drug Administration published its rule for judicious use of ABs only for treatment of animals under Veterinary Feed Directive in 2015. The limited use or ban on the use of in feed ABs increase the demand of alternatives to avoid the decline in the production performance of animals and economic losses. During last two decades, nutritionist and pharmacists have worked to develop some replacements to retain or enhance animal health and performance. Many alternatives are being used and experimentally checked for their effectiveness for animals and humans. One of the alternatives is probiotics.

Probiotics can be defined as the live microorganisms which have beneficial effects on the host when fed in adequate amounts [16]. They are considered as one of the best alternatives because of several useful aspects, both for humans and animals. They can be used to reduce the harmful bacteria and to enhance the growth and productivity of animals by improving digestion and absorption of nutrients [17]. Probiotics includes the microorganisms of different species of bacteria, fungi, or yeast. Some probiotics of bacterial origin (*Lactobacillus*, *Bacillus subtilis* [*B. subtilis*], *Bifidobacterium*, and *Streptococcus*) also have antimicrobial activity towards some pathogenic bacteria like *E. coli*, *Clostridium perfringens*, *Staphylococcus aureus*, and *Salmonella typhimurium* etc. [18]. There is need of proper selection of probiotics regarding dose and type to avoid the losses and to get valuable benefits. This article reviews the effect of probiotics (single and in combination) on growth, meat quality, bone health, eggshell quality, immunity, intestinal microflora and morphology in poultry as well as updates action mode of probioitcs.

## CONCEPT OF PROBIOTICS, COMMON VARIETIES AND SELECTION

“Probiotics could be mono or mixed cultures of the live microorganisms which have some beneficial effects on the host when fed to them in adequate amount” [16]. Angelakis, [19] stated some characteristics of a good probiotic i.e., it should not be pathogenic or toxic in nature, should have positive impact on host animal, should survive in gut environment, should be viable and remain viable under stored condition. *Bifidobacterium*, *Bacillus*, *Streptococcus*, *Lactobacillusa* and *Lactococcus* are the genera commonly used as probiotics in poultry [20]. Other commonly used probiotics are *L. casei*, *B. subtilis*, *L. acidophilus*, *L. bulgaricus*, *L. plantarum*, *L. lactis*, *Enterococcus faecalis*, *E. faecium*, *Bifidobacterium* spp., *Saccharomyces cerevisiae* (*S. cerevisiae*), and *Aspergillus oryzae*. Much work has been done on *Bifidobacterium* spp. in humans as well. *Enterococcus* spp., *Bacillus* spp., and *Saccharomyces* spp. are generally used probiotics in livestock [21]. Probiotics could be a single or multi strains and may be given in combination with other feed additives through feed or water. It is generally accepted that multiple strains probiotics are more beneficial than single stain because multiple strains have synergistic effects [22]. Probiotics exist in different forms such as granules, power, liquid, paste and gel etc. Dry form is better for storage and gastric environment [23]. Selection criteria for probiotics includes following characteristics: survival under gastrointestinal conditions, competitive exclusion of pathogenic bacteria, ability to stay/attach to mucosa of gastrointestinal tract [20] and ability to survive under different feed processing conditions [24].

## PROBIOTICS ON GROWTH PERFORMANCE AND MEAT QUALITY

Though the mechanism of probiotics to improve the growth performance is not fully understood, studies suggested that they are growth enhancers (Table 1) and ameliorate the effects of disease attack and stress. Growth performance of broilers is improved by using single and multiple strains of *Lactobacillus* spp. [25–27]. *Pediococcus acidilactici* (*P. acidilactici*) significantly improved the feed intake, weight gain (WG), and feed conversion ratio (FCR) in broilers [28]. Significant increase in WG was detected using different types of probiotics [29,30]. *L. fermentum* was reported to have various results in combination with different probiotics, no significant effect was observed with *S. cerevisiae* [31] and slight increase in body weight of broilers with *E. faecium* [32]. *B. subtilis* and *E. faecium* significantly improved final body WG and FCR [33,34]. Different strains of *B. subtilis* alone or in combination of *E. faecium* significantly improved the growth performance of broilers and layer [33,35,36]. However, contradictory results were found by using *Lactobacillus* spp. (*L. crispatus*, *L. ohnsonii*, *L. salivarius*, and some unidentified *Lactobacillus* spp.), [37]. Feeding multiple strains of probiotics (*E. faecium*, *Bifidobacterium* animals, *P. acidilactici*, *L. reuteri*, and *L. salivarius*) significantly improved WG, FCR, and production efficiency factor [38]. Many meta-analyses have also been done to evaluate the effect of probiotics on broilers and other animals. A meta-analysis was conducted by Faria-Filho et al [39] to observe the response of broilers by feeding 12 different probiotics. In this analysis, data was collected form 27 studies involving 30,146 broilers. They concluded that WG and FCR was improved by probiotics feeding. A more comprehensive meta-analysis comprising studies of 32 years were performed by Blajman et al [40]. They stated that probiotics feeding had positive effect on WG and FCR. They also suggested no difference in using single or multi-strain probiotics, but probiotics provided in water were more effective than via feed. Depending upon the health status, boilers respond differently to different probiotics. Immune modulation and balancing of gut microbiota are two possible reasons for improving growth performance of broilers by probiotics feeding. Probiotics prevent the proliferation of pathogenic bacteria by decreasing the intestinal pH through production of short chain fatty acids and maintain the equilibrium between beneficial and pathogenic bacteria which is essentially required of gut health and proper growth performance of broilers.

Variable results are found in literature about the effect of probiotics on meat quality in broilers. Improvement in meat flavor was observed by feeding broilers on *B. subtilis* and *B. licheniformis* [41] however, no significant effect of probiotics was found on sensory attributes of meat in another study [42]. Mahajan et al [43] stated that meat sensory characteristics (overall acceptability, texture, appearance, and juiciness) were positively affected by probiotics feeding. In addition, Pelicano et al [44] stated that probiotics feeding improved the meat flavor. Previous results were also supported by Ceslovas et al [45], they found improved meat quality and tenderness by probiotics. Contradictions in the results might be due to use of different type or dose of probiotics, duration of experiment or age of birds at which the probiotics were fed to the birds.

## PROBIOTICS ON BONE HEALTH AND EGGSHELL QUALITY

Previous studies confirmed that probiotics supplementation also has positive effects on bone health in poultry. Probiotics improved the gut health, enhanced the absorption capacity of intestine and bioavailability of minerals by different ways. Most of the studies presented the effect of various probiotics on tibia bone, might be since tibia is more prone to skeletal problems. *Bacillus subtilis* improved the tibia ash and ash Ca percentage [46]; *Enterococcus faecium* and lactic acid producing bacteria improved different tibia indexes including tibia Ca and Ca percentage [47]; *B. subtilis* and *B. licheniformis* improved tibia thickness, ash and P percentage [48]; *Lactobacillus sporogenes* enhanced tibia breaking strength and ash percentage [49] whereas *Clostridium butyricum* and *B. subtilis* also enhanced tibia breaking strength, ash contents and tibia weight/length index [50] in broilers. Additionally, probiotics also have the ability to protect the bone under different pathological conditions as described by Sadeghi [46], that supplementation of *B. subtilis* in broilers challenged with *Salmonella enteritidis* (*S. enteritidis*) improved the tibia bone minerals contents including Ca at 21 days of age. Probiotics also reduce the negative effects of low Ca diet in broilers [50]. Another study indicated that supplementation of *B. subtilis* in diet of laying hens (at 0.5 or 1.0 g/kg of feed) boosted the tibia indexes such as length, weight, density, and ash contents [51]. Similarly, *B. subtilis* feeding in laying hens also decreased the number of unmarketable eggs [52]. Furthermore, different probiotics also improved the eggshell quality in laying hens [53].

**Figure 1:**
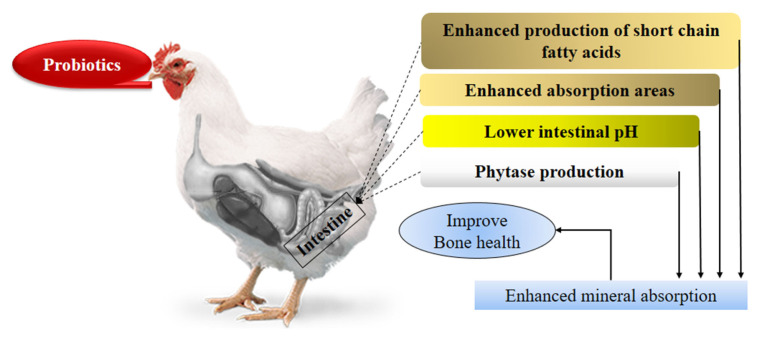
Mechanisms underlying improved bone health by probiotics feeding.

Basic reason underlying improved bone health by probi otics feeding is enhanced Ca absorption by different ways (Figure 1). An *in vitro* study reported that *L. salivarius* enhanced trans-epithelial Ca transport in human cells, while *in vivo* study supported this result as Ca and P concentration was higher in probiotics supplemented rats than control group [54]. Probiotics increase Ca bioavailability through different ways such as they produce phytase having ability to degrade phytate and release bounded minerals like Ca and P [55]. As mentioned in another section that probiotics positively affect the gut morphology and increase the absorption area of intestine by increasing villus length and villus length to crypt depth ratio. So, higher absorption area increases the rate of minerals absorption. Another possible way of increasing mineral absorption is reduced intestinal pH due to short chain fatty acids produced by probiotics. Acidic environments favor the absorption of Ca [56].

## PROBIOTICS ON IMMUNE RESPONSE

Probiotics as an alternative to ABs are considered to improve the health status and immunity in poultry birds (Table 2). Bai et al [31] demonstrated that T cell immune system was improved by probiotics (combined: *L. fermentum* and S*. cerevisiae*) without compromising the growth performance of broilers. It is also proved that *Lactobacillus* (*LAB*) influenced the chemokine gene expression and cytokines production in chicken [57]. *LAB* improved the production of anti- and pro-inflammatory cytokines in the intestinal epithelium of broiler chicken. The cytokines production influences the overall immune system. Increase in antibody production by β-lymphocytes is another potential mechanism of *LAB* in improving the immunity in broiler. *L. fermentum* improved the blood CD4^+^ lymphocyte value as well as interferon-γ and tumor necrosis factor-α expression in the ileum. Waititu et al [58] fed a mixture of three *Bacillus subtilis strains* (BSS) and *Propionibacterium acidipropionici* (PA) to broilers and found downregulation of ileal expression of different cytokines by PA and interleukin-10 (IL-10) in the spleen, IL-2, IL-4, IL-6, and toll like receptor-2b in all examined tissues by BSS, whereas IL-13 was upregulated in spleen by BSS. Kabir et al [30] found significant increase in the antibodies concentration and weight of immune organs (bursa and spleen) of broilers by using Protexin Boost (a mixture of different probiotics) at the rate of 0.2 g/liters of drinking water.

*E. faecium* improved immunoglobulin A production in layers [59]. Similarly, findings of Haghighi et al [60] also suggested the enhanced systemic immunity and local antibodies production in broilers at 14 days post hatching by feeding *LAB* in combination with *B. bifidum* and *S. faecalis*. *LAB* also increase intestinal intraepithelial lymphocyte expression of the surface markers CD3^+^, CD4^+^, and CD8^+^ in broilers [61]. *L. fermentum* (10^7^ cfu/g) with *S. cerevisiae* (2× 10^6^ cfu/g) significantly increased CD8^+^, CD3^+^, and CD4^+^ levels in broilers [31]. Hatab et al [33] found improved antibody production against Newcastle disease virus in layers birds by *B. subtilis* and *E. faecium*. Similarly, *B. subtilis* feeding increased the antibody titers levels in broilers against Newcastle disease, Infectious bronchitis, and Infectious bursal disease virus [35].

## PROBIOTICS ON INTESTINAL MICROBIOFLORA AND MORPHOLOGY

**Figure 2:**
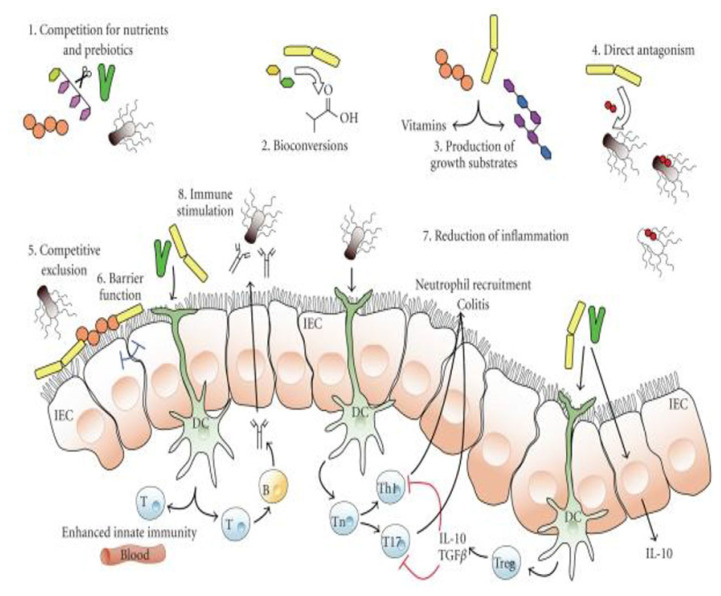
Diagram showing the potential impact of probiotic bacteria on microbiota. The schematic diagram showing mechanism: 1) competition for nutrients and prebiotics for growth, 2) bioconversion of nutrients into other substances with selective inhibitory properties against pathogens, 3) production of growth substances like vitamins for other bacterial organisms, 4) direct antagonism through antibacterial agents like bacteriocins, 5) competitive exclusion for binding sites, 6) barrier function, 7) lessening of inflammation, hence changing intestinal ecosystem for colonization, and 8) modulation of innate immunity. IEC, epithelial cells; T, T-lymphocytes; B, B-lymphocytes; DC, dendritic cells; T, T-cells; Th1, T helper type 1; T17, a subset of T helper cell that produce interleukin 17; Treg, T-regulatory cell; IL-10, interleukin 10; TGFβ, transforming growth factor beta (adopted from https://www.customprobiotics.com/mechanisms-of-action ).

Animal health status and growth performance are directly linked with gut health and its microflora. A healthy gut is more defensive against pathogenic microorganisms and works more efficiently for nutrients absorption. A stable microflora is also required to avoid infections in the gut which helps the animals in different ways, by avoiding the colonization of pathogens by bacterial antagonism or occupying the attachment site in gut and interfering with bacterial activities. The intestinal microflora is quite stable but influenced by different environmental factors and health status of animals. Environmental factors include diet, hygiene conditions and stress. The most important factor which affects the gut microflora is diet. Probiotics are generally applied to regulate the gut microbiota [62] (Figure 2). Similarly, gut morphological parameters also have significant importance towards health and performance of poultry birds and animals as higher villus length to crypt depth ratio with higher villus height is directly linked to increased absorption area and nutrient absorption. In addition, goblet cell concentration in intestinal villi is another gut health parameter because these cells reduce the chances of attachment of pathogenic bacteria to intestinal epithelium and enhance mucin production [63]. Probiotics have positive effects on gut histomorphology, but the degree of effectiveness may vary from strain to strain. As increase in villus height with decrease in crypt depth was observed in broilers fed probiotic containing *L. acidophilus*, *L. casei*, E*nterococcus faecium*, and *Bifidobacterium thermophilum* [17]. Similarly, supplementation of *B. coagulans* [64]; combination of *L. acidophilus*, *L. casei*, *Enterococcus faecium* and *Bifidobacterium thermophilum*, [65]; *L. reuteri* and *L. salivarius* [66]; *Propionibacterium acidipropionici* [67]; mixture of *B. licheniformis*, *B. subtilis* and *S. cerevisiae* [68] and *P. acidilactici* [69] in broilers exhibited positive effect on gut histomorphology with increased villus length and villus length to crypt depth ration which suggested that probiotics enhanced nutrient absorption.

Hayashi et al [70] fed *B. subtilis* to broilers and found improved histologic modification associated with stimulation of the defense reaction in the ileum. Most of the *LAB* species affect the gut health positively. Chen et al [71] found significant reduction in *Salmonella* counts recovered from the cecal tonsils, spleens, and livers and Gao et al [25] observed accelerated maturation of intestinal microbiota by *LAB* feeding in broilers. *LAB* may be efficacious for reduction of *S. enteritidis* in young chicken [72]. Increased anaerobic bacteria in the ileum and caeca, and lactic acid bacteria and *lactobacilli* in the caeca with significant increase in weight of small intestinal (jejunum and ileum) was observed with *LAB*. Probiotics also reduced the count of pathogenic bacterial [37]. Palamidi et al [38] suggested that *LAB* increased the digestibility of dry matter and fat in diet. *LAB* significantly increased the number of beneficial bacteria one-week post feeding and reduced the count of pathogenic bacterial by 40 days post feeding in broilers [27]. It is suggested by researches that probiotic feeding increase the total count of beneficial bacteria in intestine and reduce the number of disease-causing bacteria [73,74]. Supplementation of *B. subtilis* alone or in combination with *S. boulardii* have a significantly positive effect on intestinal histopathology and microflora in broilers [34,35] (Table 3).

## UPDATED ACTION MODE OF PROBIOTICS

**Figure 3:**
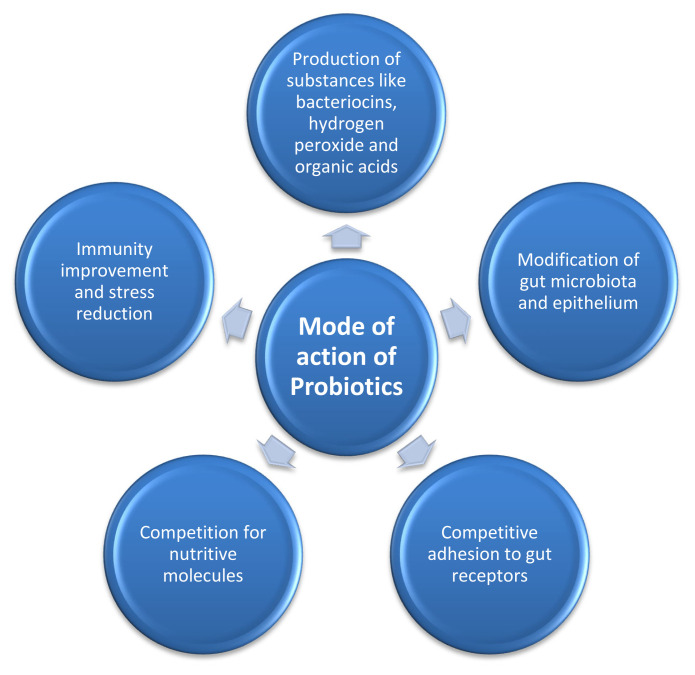
Showing mode of action of probiotics that results improved production performance in monogastric animals by producing many gut microbiota modulating factors (like bacteriocins, organic acids etc) that improve immunity, reduce stress, improve nutrient production and utilization and modulate competitive adhesion to gut.

There are different mechanisms through which probiotics work to facilitate the host animals, which may include occupation of epithelial cells to avoid the colonization of pathogenic bacteria, stimulation of enzyme synthesis, production of some antimicrobial substances and reduction of toxin production, boost up the immunity and stress reduction [75] (Figure 3).

Occupation of the site of attachment in GIT by probiotics to avoid pathogenic bacteria could result in physical blocking of pathogen colonization. This physical blocking alters the environment of GIT and is beneficial for host animals to improve their immune system. This mechanism was found by Nurmi and Rantala [76], suggesting that *Salmonella* colonization in the GIT of newly hatched chicken could be reduced by feeding a suspension of gut contents of healthy adult chicken. In this way pathogenic bacteria are excluded from the site of replication. Probiotics also produce some antimicrobial substances like bacteriocins, hydrogen peroxide and organic acids [77]. Organic acids decrease the pH of intestine which helps absorption of minerals (calcium, iron, copper, magnesium, and manganese) and protein. Probiotics help to regenerate intestinal mucosa, upregulate the mucous production and intestinal motility, modulate host immune system by stimulation of antibody production and natural killer cells [77], and improve the digestion by increasing the secretions of digestive enzymes. Probiotics can also reduce the blood cholesterol level by virtue of their bile salt hydrolase activity which lower the chances of cardiovascular disease [78]. There is another mode of action in probiotics called cross feeding, which means feeding of one bacteria is beneficial for another bacteria production. For example, lactic acid produced by lactic acid producing bacteria can be used by butyric acid producing bacteria and produce a large amount of butyric acid [79]. The cross-feeding mechanism of butyric acid production is beneficial to enhance the growth performance.

## CONCLUSION

Keeping in view the past studies, it could be concluded that probiotics could be serve as growth promoters because they improve the gut health which ultimately improves the nutrient digestibility by enhancing the digestive enzyme activities and improves the growth performance and meat quality. Probiotics also modulate the immune response in such a way to protect host animals from pathogenic disease, in addition through competitive exclusion mechanism they protect the host animals from colonization by pathogenic bacteria. Inconsistency in results could be due to numerous biotic and abiotic factors which could be resolved by further studies concerning detail of mode of action, mode of delivery and improving probiotic *in vivo* efficacy.

## Figures and Tables

**Table 1:** Effect of probiotics on growth performance of poultry

Sr	Probiotic	Animal	Inclusion level	Duration (d)	Growth performance	Reference
1	*L. plantarum strain IMAU10120 (LP-8)*	Broiler	2×10^6^ cfu/mL	42	Improved FCR	[25]
2	*L. fermentum* (*CIP 102980*) or a strain of *L*. spp. (*Autruche 4; DQ418552*)	Broiler	Dose of 1 mL containing 10^7^ cells	29	WG and feed conversion efficiency was significantly improved	[26]
3	*L. agilis JCM 1048* and *L. salivarius subsp. salicinius JCM 1230*	Broiler		40	Significant increase in weight gain	[27]
4	*Pediococcus acidilactici MA18/5M*	Broiler	0.8 to 1.6 g	42	Significant improvement in overall growth performance	[28]
5	*3 Bacillus subtilis strains*	Broiler	3×10^5^ cfu/g of finished feed	41	Improved overall body weight gain	[29]
6	*A. oryzae, B. bifidum, B. thermophilus, C. pintolopessi, E. faecium, L. acidophilus, L. bulgaricus, L. plantarum* and *L. rhamnosus*	Broiler	2 gm/10 liters drinking water	42	Significant increase in WG, breast and leg meat yield	[30]
7	*L. fermentum* and *S. cerevisiae*	Broiler	0.1% to 0.2%	42	No significant effect on growth performance	[31]
8	*L. fermentum CCM 7158*	Broiler	1×10^9^ cfu/kg	42	Slightly increased body weight	[32]
	*E. faecium M 74*		2×10^9^ cfu/kg			
9	*B. subtilis* and *E. faecium*	Layer	1 and 2 g/kg of feed	70	Significant effect on final body weight gain and FCR	[33]
10	*S. boulardii* and *B. subtilis B10*	Broiler	1×10^8^ cfu/kg of feed	72	Significant improvement in live body weight	[34]
11	*B. subtilis C-3102*	Broiler	0.1% of diet	42	Significantly increased body weight and decreased FCR	[35]
12	*B. subtilis PB6 (ATCC-PTA 6737)*	Broiler	5×10^11^ cfu/kg feed	35	FCR was significantly improved	[36]
13	*L. ohnsonii, (No.709) L. crispatus (No.697) L. salivarius (No.461)* and *unidentified Lactobacillus* spp.	Broiler		42	No significant effect on growth performance	[37]
14	*L. reuteri (DSM 16350), E. faecium (DSM 16211), Bifidobacterium animals (DSM 16284), P. acidilactici (DSM 16210)* and *L. salivarius (DSM 16351)*	Broiler	10^8^ cfu/kg diet	42	Probiotic addition improved WG, FCR and production efficiency factor	[38]
15	*Propionibacterium acidipropionici*	Broiler	7.5×10^4^ cfu/g of feed	21	Significant improvement in FCR	[58]
16	*B. subtilis*	Broiler	250 g/ton	21	Improved animal performance	[70]

FCR, feed conversion ratio; WG, weight gain; cfu, colony forming unit.

**Table 2:** Effect of probiotics on immune response of poultry

Sr	Probiotic	Animal	Inclusion level	Duration	Immune response	Reference
1	*L. plantarum strain IMAU10120*	Broiler	2×10^6^ cfu/mL	42 days	Induced the highest level of immunity response	[25]
2	*A. oryzae, B. bifidum, B. thermophilus, C. pintolopessi, E. faecium, L. acidophilus, L. bulgaricus, L. plantarum* and *L. rhamnosus*	Broiler	2 gm/10 liters of drinking water	42 days	Significantly enhance the antibodies production and weight of immune organs	[30]
3	*L. fermentum S. cerevisiae*	Broiler	10^7^ cfu/g 2×10^6^ cfu/g	42 days	Significant increase in CD3^+^, CD4^+^ and CD8^+^ levels	[31]
4	*B. subtilis* and *E. faecium*	Layer	1 to 2 g/kg of feed	10 weeks	Improve antibody levels against Newcastle disease virus	[33]
5	*S. boulardii* and *B. subtilis B10*	Broiler	10^8^ cfu/kg of feed	72 days	Significant improvement in inflammatory and anti-inflammatory cytokines, IgA+ cells in jejunum, intestinal cytokines IL-6, tumor necrosis factor-α, IL-10 and transforming growth factor-β	[34]
6	*B. subtilis C-3102*	Broiler	0.1% of diet	42 days	Increase the ND, IB, and IBD titers levels	[35]
7	*L. reuteri (DSM 16350), E. faecium (DSM 16211), B. animals (DSM 16284), P. acidilactici (DSM 16210)* and *L. salivarius (DSM 16351)*	Broiler	10^8^ cfu/kg diet	42 days	Induced an anti-inflammatory response at cecal level	[38]
8	Blend of *3 Bacillus substilis strains (BSS)* and *Propionibacterium acidipropionici* (PA)	Broiler	7.5×10^4^ cfu/g	21 days	*PA* downregulated ileal expression of TLR-2b, IL-2, IL-4, IL-6, IL-10, and IL-13, whereas *BSS* downregulated TLR-2b, IL-2, IL-4, and IL-6 in all 3 tissues	[58]
9	*E. faecium*	Layer	4×10^8^ cfu/kg	48 days	Improvement in IgA production	[59]
10	*L. acidophilus, B. bifidum* and *S. faecalis*	Broiler	10^6^ bacteria/chick on day of hatching	14 days	Enhanced systemic and local antibodies production	[60]
11	*Lactobacillus* spp.	Broiler	1 g/kg feed	35 days	Increase intestinal intraepithelial lymphocyte expressing the surface markers CD3, CD4 and CD8	[61]
12	*L. acidophilus, L. fermentum L. plantarum* and *E. faecium (A) L. jensenii, L. plantarum, L. fermentum* and *L. casei, (B)*	Broiler	0.2 mL/chick, 10^9^ cfu/mL	1 to 3 days of chick’s life	Group (A) decrease IL-1β, IL-6, IFN-γ and improve IL-10 levels in the cecal tonsils compared with (B)	[71]

cfu, colony forming unit; CD, cluster of differentiation molecules; Ig, immunoglobulin; IL, interleukin; ND, Newcastle disease; IB, Infectious bronchitis; IBD, Infectious bursal disease ; IgA, immunoglobulin A; PA, *Propionibacterium acidipropionici*; TLR, toll like receptor; IFN, interferon.

**Table 3:** Effect of probiotics on gut health and microbiota of poultry

Sr	Probiotic	Animal	Inclusion level	Duration	Gut health and microbiota	Reference
1	*L. plantarum strain IMAU10120*	Broiler	2×10^6^ cfu/mL	42 days	Accelerated maturation of intestinal microbiota, and stimulated the growth of many intestinal *Lactobacillus* spp.	[25]
2	*L. agilis JCM 1048* and *L. salivarius subsp. salicinius JCM 1230*	Broiler	10^7^ cfu per gram of feed	40 days	Increase the population of beneficial microflora and maintain the natural balance of microbes in gut	[27]
3	*S. boulardii* and *B. subtilis B10*	Broiler	1×10^8^ cfu/kg of feed	72 days	Significant improvement in intestinal villus height, width, and number of goblet cells	[34]
4	*B. subtilis C-3102*	Broiler	0.1% of diet	42 days	Decreased crypt depth and increased villus height with significant reduction in *E. coli*, *coliform*, and *Salmonella* populations of ceca	[35]
5	*B. subtilis PB6 (ATCC-PTA 6737)*	Broiler	5×10^11^ cfu/kg feed	35 days	Significant reduction in intestinal *C. perfringens* counts, improved villi length and villi length to crypt depth ratio	[36]
6	*L. johnsonii, (No.709) L. crispatus (No.697) L. salivarius (No.461)* and *unidentified Lactobacillus* spp.	Broiler		6 weeks	All probiotics tended to reduce the number of *Entero-bacteria* in the ileum	[37]
7	*L. reuteri (DSM 16350), E. faecium (DSM 16211), B. animals (DSM 16284), P. acidilactici (DSM 16210)* and *L. salivarius (DSM 16351)*	Broiler	10^8^ cfu/kg diet	42 days	Significant improvement in total tract apparent digestibility of crude protein, fats and ileal digestibility of dry matter	[38]
8	Blend of 3 *Bacillus substilis strains*	Broiler	7.5×10^4^ cfu/g	21 days	Both reduced the apparent ileal digestibility of dry matter and crude protein	[58]
	*P. acidipropionici* (*PA*)					
9	*Lactobacillus* spp.	Broiler	1 g/kg feed	35 days	Increased the birds’ resistance to *Eimeria acervuline*	[61]
	*B. subtilis*	Broiler	250, or 500 g/ton	21 days	Higher dose improves the defense response in the ileum	[70]
10	*L. acidophilus, L. fermentum L. plantarum* and *E. faecium*	Broiler	0.2 mL/chick, 10^9^ cfu/mL	1–3 days of chick’s life	*Salmonella* counts recovered from the cecal tonsils, spleens were significantly reduced	[71]
11	*Lactobacillus based probiotic culture* (*FM-B11*)	Broiler	10^4^, 10^6^, and 10^8^ cfu/chick	1 h after administration	Higher dose significantly reduced *Salmonella Enteritidis* population	[72]
12	*A. oryzae, B. bifidum, L. casei, L. acidophilus, Torulopsis spp*. and *S. faecium*	Broiler	100 mg/kg diet	35 days	Decrease the population of *Campylobacter* and *Coliform* in the gut	[73]
13	*L. salivarius*	Broiler	10^8^ cfu 100 μL of phosphate-buffered saline	14 days	Lower the number of *C. perfringens* and *S. enteritidis* in the gut	[74]

cfu, colony forming unit.
